# Transcriptome dataset of *Babesia bovis* life stages within vertebrate and invertebrate hosts

**DOI:** 10.1016/j.dib.2020.106533

**Published:** 2020-11-17

**Authors:** Massaro W. Ueti, Wendell C. Johnson, Lowell S. Kappmeyer, David R. Herndon, Michelle R. Mousel, Kathryn E. Reif, Naomi S. Taus, Olukemi O. Ifeonu, Joana C. Silva, Carlos E. Suarez, Kelly A. Brayton

**Affiliations:** a Animal Disease Research Unit, USDA-ARS, Pullman, WA, United States; b Program in Vector-borne Disease, Department of Veterinary Microbiology and Pathology, Washington State University, Pullman, WA, United States; c Paul G. Allen School for Global Animal Health, Washington State University, Pullman, WA, United States; d Institute for Genome Sciences, University of Maryland School of Medicine, Baltimore, MD, United States; e Department of Microbiology and Immunology, University of Maryland School of Medicine, Baltimore, MD, United States

**Keywords:** Bovine babesiosis, *Babesia*, Bovine, Gene expression, Kinetes, *Rhipicephalus microplus*

## Abstract

*Babesia bovis* is a hemoprotozoan parasite of cattle that has a complex life cycle within vertebrate and invertebrate hosts. In the mammalian host, *B. bovis* undergoes asexual reproduction while in the tick midgut, gametes are induced, fuse, and form zygotes. The zygote infects tick gut epithelial cells and transform into kinetes that are released into the hemolymph and invade other tick tissues such as the ovaries, resulting in transovarial transmission to tick offspring. To compare gene regulation between different *B. bovis* life stages, we collected parasites infecting bovine erythrocytes and tick hemolymph. Total RNA samples were isolated, and multiplexed libraries sequenced using paired-end 100 cycle reads of a HiSeq 2500. The data was normalized using the TMM method and analysed for significant differential expression using the generalized linear model likelihood ratio test (GLM LRT) in edgeR. To validate our datasets, ten genes were selected using NormFinder. Genes that had no significant fold change between the blood and tick stages in the RNA-Seq datasets were tested by quantitative PCR to determine their suitability as “housekeeping” genes. The normalized RNA-Seq data revealed genes upregulated during infection of the mammalian host or tick vector and six upregulated genes were validated by quantitative PCR. These datasets can help identify useful targets for controlling bovine babesiosis.

## Specifications Table

**Table:** 

Subject	Parasitology
Specific subject area	Transcriptome analysis of *Babesia bovis* life stages.
Type of data	Tables Graphic Excel file
How data were acquired	Illumina HiSeq™ 2500 Bio-RAD CFX96™ Real-Time PCR
Data format	Raw Analysed
Parameters for data collection	*Babesia bovis* blood stages were collected from bovines with acute parasitemia and suspended in TRIzol (Thermo Fisher Scientific, Waltham, MA). *Babesia bovis* kinetes were collected from infected replete female ticks by extraction via pressurized capillary tubing, pooled, concentrated, and suspended in TRIzol. Total RNA was extracted, and library construction performed according to Illumina TruSeq mRNA library protocols.
Description of data collection	Counts were generated from alignments for each gene using the Subread feature of Counts v1.6.0. Genes without at least 1 read per million mapped reads across all three samples within a group were removed, data was normalized using the TMM method, and analysed for differential expression significance testing using the generalized linear model likelihood ratio test (GLM LRT) method in edgeR v3.20.9. The false discovery rate (FDR) method was employed to correct for multiple testing and genes were termed significantly differentially expressed if their logFold Change (logFC) value was greater than or equal to 1 and the FDR set to 5%. Triplicate RNA samples from *B. bovis* blood or kinete stages were used for qPCR. Quantitative PCR was performed in triplicate using a Bio-RAD CFX96™ Real-Time PCR Detection System. The transcript level of six genes of interest was normalized by dividing the transcript level of the gene of interest with that of the housekeeping genes. To identify suitable “housekeeping” genes, Excel (Microsoft Office 2013) with NormFinder and the comparative delta-Ct method from the RNA-Seq dataset were used to select ten genes that had no significant fold changes between the blood and kinete stages. The top five housekeeping genes were then used to normalize transcript levels in the qPCR data.
Data source location	The U.S. Department of Agriculture-ARS-Animal Disease Research Unit. Pullman/Washington State United States of America 46.730873 ° N, -117.163475 ° E
Data accessibility	Raw data were deposited at the NCBI Gene Expression Omnibus under accession number GSE144066. Direct URL to data: https://www.ncbi.nlm.nih.gov/geo/query/acc.cgi?acc=GSE144066.
Related research article	M.W. Ueti, W.C. Johnson, L.S. Kappmeyer, D.R. Herndon, M.R. Mousel, K.E. Reif, N.S. Taus, O.O. Ifeonu, J.C. Silva, C.E. Suarez, K.A. Brayton. Comparative analysis of gene expression between *Babesia bovis* blood stages and kinetes allowed by improved genome annotation. Int. J. Parasitol. doi: 10.1016/j.ijpara.2020.08.006.

## Value of the Data

• Comparison between life stages can help identify useful targets for therapeutic intervention and vaccine development to control bovine babesiosis or block parasite infection of the tick vector.
• These data can benefit scientists at universities, federal agencies, and international institutions working towards prevention of *B. bovis*.
• These datasets may be the foundation to define potential targets for understanding transmission mechanisms of tick-borne protozoan parasites of humans and animals.
• The kinete dataset contains reads for *R. microplus* hemocytes that could be used to elucidate tick responses to *Babesia* infection.

## 1 Data Description

**Fig. 1:**
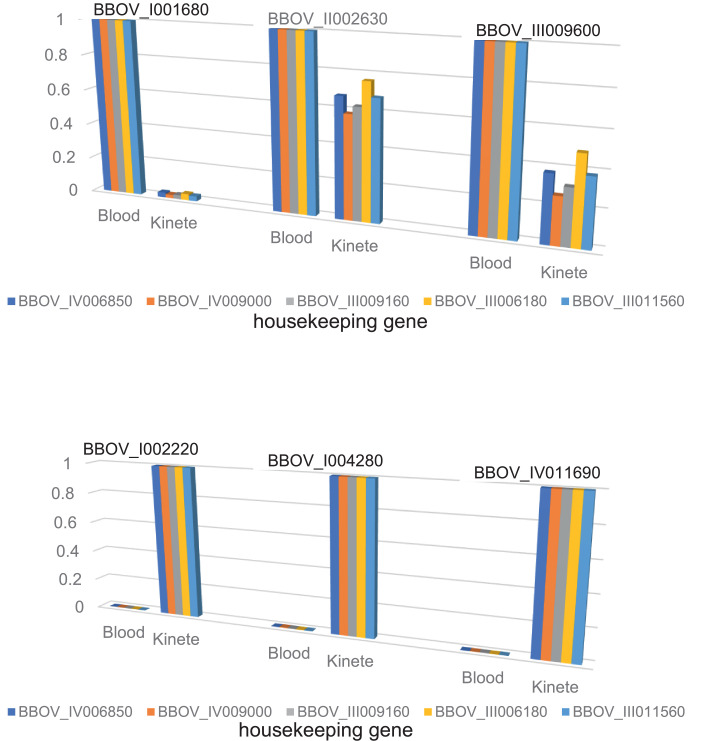
Ratios of housekeeping gene relative expression as compared to the test genes. The top panel are genes upregulated in *B. bovis* blood stages and the bottom panel are genes upregulated in kinetes.

Tables 1–4 show differential expression of gene families by *B. bovis* blood stages or kinetes, including SMORFs, SBPs, GCC2 and GCC3 domain-containing proteins, and VESAs. Normalized data were used to determined fold increase by dividing normalized counts of one stage with the other. Table 5 indicates differential gene expression associated with elements of the *B. bovis* glideosome between blood stages and kinetes. To validate the RNA-Seq, three test genes from either kinete stage (BBOV_I002220, BBOV_I004280, BBOV_IV011690) or blood stages (BBOV_I001680, BBOV_II002630, BBOV_III009600) were selected based on their magnitude of transcription. A panel of housekeeping genes (BBOV_IV006850, BBOV_IV009000, BBOV_III009160, BBOV_III006180, BBOV_III011560) demonstrates a consistent pattern of gene expression differences between *B. bovis* blood stages and kinetes. Fig. 1 illustrates the consistent ratios of housekeeping gene relative expression as compared to the test genes. Supplementary Table S1 is an Excel Spreadsheet with normalized RNA-Seq data and analyzed in the context of differential gene expression between *B. bovis* blood stages and kinetes. Table S1 contains *B. bovis* gene identifiers, Log Fold Change, Log Count per Million Reads, False Discovery Rate, triplicate reads for *B. bovis* blood stages and kinetes (normalized and raw counts).

**Table 1:** Differential expression of the small open reading frame (*smorf*) gene family between *B. bovis* blood stages and kinetes.

GeneID	norm blood stages	norm kinetes	Fold increase in blood stages	Fold increase in kinetes
BBOV_III011930	2113.913468	14.36620151	147.1449127	0.006796022
BBOV_III001320	536.3841036	2.982448672	179.8468851	0.005560285
BBOV_I003880	518.6261427	7.285320965	71.18782345	0.014047346
BBOV_III007740	398.7738069	110.4001374	3.612077088	0.276849019
BBOV_II004160	380.0745053	2.545392465	149.3186259	0.006697088
BBOV_II004220	369.9142849	2.877928198	128.534925	0.007779987
BBOV_I003890	288.1595966	2.645425925	108.9274864	0.009180419
BBOV_IV007930	286.4893288	1.926687667	148.6952627	0.006725164
BBOV_II000060	280.9586624	1.984377164	141.5853133	0.007062879
BBOV_III000050	240.2349071	1.13933101	210.8561122	0.004742571
BBOV_I001120	222.2547257	36.4316058	6.10060196	0.16391825
BBOV_II006810	208.0321558	5.086740939	40.89694331	0.024451705
BBOV_IV006430	207.1816437	0.937498517	220.9941028	0.004525008
BBOV_I001180	202.7519971	1.332583402	152.1495741	0.00657248
BBOV_II002275	194.4475113	11.84410832	16.41723515	0.060911596
BBOV_IV007970	187.8401442	0.724434865	259.2919714	0.003856656
BBOV_II001390	169.3409791	10.7185897	15.79881158	0.0632959
BBOV_IV006390	126.4761588	12.76775186	9.905906709	0.100949871
BBOV_II002280	109.3296517	3.105479146	35.20540521	0.028404729
BBOV_I001370	108.4269976	0.615054735	176.2883713	0.005672524
BBOV_IV000090	99.97305676	0.97946984	102.0685402	0.009797338
BBOV_III011960	97.89184227	1.043228584	93.8354679	0.010656951
BBOV_II007780	80.91934755	0.623937094	129.6915159	0.007710605
BBOV_III000020	77.72837628	0.441915862	175.8895368	0.005685387
BBOV_IV012140	69.65059029	2.152564713	32.35702503	0.03090519
BBOV_III007710	66.79064133	32.61366171	2.047934449	0.488296879
BBOV_IV000040	66.34182859	3.766155266	17.61526647	0.05676894
BBOV_III002350	63.3051779	3.209907951	19.72180476	0.050705299
BBOV_I003860	61.03939933	1.178418838	51.79771178	0.019305872
BBOV_I001160	56.8016851	2.876162626	19.74912148	0.050635164
BBOV_III002340	51.26993531	0.410873441	124.7827924	0.008013925
BBOV_I001420	45.33252697	0.838674649	54.05257813	0.018500505
BBOV_I001170	43.73955786	0.743409174	58.83645158	0.016996266
BBOV_II007820	32.65136192	0.406850737	80.25390869	0.012460452
BBOV_II004150	31.64665067	0.486700297	65.02287115	0.015379204
BBOV_IV007960	29.20461566	0.486700297	60.00533771	0.016665184
BBOV_II002290	25.70031072	11.88324468	2.162735129	0.462377471
BBOV_II006800	16.08857604	607.0183122	0.026504268	37.72977241
BBOV_II001380	13.69017871	2.146657517	6.377439625	0.156802739
BBOV_II000400	8.75589443	406.4511482	0.021542305	46.42028881
BBOV_I005150	4.467630153	3.39732353	1.315044067	0.760430791
BBOV_I003850	3.639805192	0.281682051	12.92167951	0.077389321
BBOV_IV006420	1.559208382	4.05065043	0.384927905	2.59788908
BBOV_III000690	0.288793277	92.75983833	0.003113344	321.198053

**Table 2:** Differential expression of the spherical body protein gene family between *B. bovis* blood stages and kinetes.

GeneID	norm blood stages	norm kinetes	Fold increase in blood stages	Fold increase in kinetes
BBOV_I004210	9564.525657	716.5825508	13.34741635	0.074920867
BBOV_IV005390	4059.333716	31.38524799	129.3389084	0.007731625
BBOV_II000680	3355.546841	21.10031815	159.0282581	0.006288191
BBOV_II000740	1058.592079	23.79627366	44.48562387	0.022479172
BBOV_III005860	679.0452333	5.850819611	116.0598478	0.008616244
BBOV_III005600	384.621592	4.203584114	91.49848831	0.010929142
BBOV_III006540	289.7373304	8.541515569	33.92106799	0.029480204
BBOV_III005830	252.4609668	4.173006004	60.498587	0.016529312
BBOV_III006460	239.8983689	413.0159404	0.580845303	1.721628798
BBOV_III005840	162.7218894	12.47769378	13.04102282	0.076681102
BBOV_III006520	50.57652699	0.628796749	80.43382394	0.012432581
BBOV_III005630	36.93756706	1.347535007	27.41121148	0.036481423
BBOV_III005790	19.96872969	3.655263286	5.463007211	0.183049365
BBOV_III006480	18.77035174	0.901596442	20.81901711	0.048033007
BBOV_III006500	4.383303317	0.20015859	21.89915163	0.045663869

**Table 3:** Differential expression of the GCC2 and GCC3 domain containing protein gene family between *B. bovis* blood stages and kinetes.

GeneID	norm blood stages	norm kinetes	Fold increase in blood stages	Fold increase in kinetes
BBOV_IV006260	319.5826078	22.37882551	14.28057999	0.070025167
BBOV_III011740	197.335074	35.38007157	5.577577016	0.179289322
BBOV_IV006250	4.042298771	4387.570389	0.000921307	1085.414671
BBOV_III011730	3.208575878	2445.955909	0.001311788	762.3182379

**Table 4:** Differential expression of the variant erythrocyte surface antigen (*ves1*) gene family between *B. bovis* blood stages and kinetes.

GeneID	norm blood stages	norm kinetes	Fold increase in blood stages	Fold increase in kinetes
BBOV_III000015	1.728967521	0.601312722	2.875321706	0.34778717
BBOV_III001300	1.02316527	0.401991082	2.545243701	0.392889687
BBOV_II002310	11.46011732	4.90734948	2.335296756	0.428211103
BBOV_I001190	1.921214622	0.906828737	2.118608006	0.472008034
BBOV_IV007950	1.919285603	0.94926437	2.021866261	0.494592555
BBOV_I005825	156.5772135	79.50478851	1.969406075	0.507767297
BBOV_II002320	423.6835178	220.0983315	1.924973783	0.519487594
BBOV_IV005670	331.7736784	174.558981	1.900639408	0.526138728
BBOV_II000380	41.83335358	22.80774353	1.834173272	0.545204761
BBOV_IV005660	140.5719566	82.52085286	1.703471932	0.587036382
BBOV_II000040	16.7797131	10.55649865	1.589515014	0.629122714
BBOV_III001270	24.07677147	15.19922865	1.58407851	0.631281842
BBOV_II007810	7.620384667	4.871933002	1.564139873	0.639329012
BBOV_II007830	3.44796873	2.208278049	1.561383419	0.640457679
BBOV_III001150	18.99083241	12.39033947	1.532712841	0.652437934
BBOV_I000060	3.967674309	2.651030862	1.496653383	0.668157378
BBOV_I000070	10.11356796	7.128294751	1.418792055	0.704824922
BBOV_IV002830	23.37186412	16.55999767	1.41134465	0.708544153
BBOV_III002370	12.47428832	8.974695799	1.389939959	0.719455537
BBOV_II000900	24.01138202	22.98856169	1.044492576	0.957402688
BBOV_I001410	10.98244456	12.27491138	0.894706627	1.117684803
BBOV_II000030	6.160960597	7.564935187	0.814410229	1.227882417
BBOV_III011940	26.85581707	36.03944587	0.745178413	1.341960506
BBOV_I000050	34.99932496	48.47120721	0.722064231	1.384918345
BBOV_II000945	8.125332914	12.97149013	0.626399344	1.596425681
BBOV_III007700	19.30319549	31.04248606	0.621831494	1.608152706
BBOV_II002260	0.539728944	0.905991786	0.595732712	1.678605154
BBOV_IV000030	14.53940728	24.77284468	0.586909072	1.703841443
BBOV_II006820	14.94653581	29.50277234	0.50661462	1.973886975
BBOV_III007110	420.2763021	50.88074443	8.260026594	0.121064986
BBOV_III007140	563.2564757	77.88629427	7.231779108	0.13827856
BBOV_III006920	478.708575	140.9323883	3.396725059	0.29440122
BBOV_III003060	250.1297463	78.89379732	3.170461491	0.315411495
BBOV_III006080	798.0562832	5.063209234	157.6186656	0.006344426
BBOV_IV001490	499.5313164	5.215861947	95.77157554	0.010441511
BBOV_IV007980	128.4011235	2.044485845	62.80362559	0.015922648
BBOV_III011950	53.41646004	1.155794466	46.21622756	0.021637422
BBOV_I001320	4.013872157	0.104520474	38.40273571	0.026039812
BBOV_I005865	193.6617495	5.222233452	37.08408505	0.026965746
BBOV_III002320	44.98541218	1.343047993	33.49501463	0.029855189
BBOV_I000010	32.39996069	1.048088238	30.91339021	0.032348442
BBOV_III003090	4982.64518	189.2864097	26.32331179	0.037989141
BBOV_III000090	8.739195077	0.401154131	21.78513044	0.04590287
BBOV_I005120	165.8736178	9.685269174	17.12638181	0.058389449
BBOV_I005835	90.24457916	5.64889545	15.97561505	0.062595399
BBOV_IV000350	201.2939525	17.46459112	11.52583253	0.086761628
BBOV_I001340	6.804356674	0.614217784	11.07808476	0.090268311
BBOV_IV002840	1937.126989	201.8771272	9.595574376	0.10421471
BBOV_II004130	16.85364966	2.155539876	7.818760323	0.127897513
BBOV_I005945	68.75377453	9.111370654	7.545931028	0.132521752
BBOV_I005905	96.32245181	13.5837803	7.090990116	0.14102403
BBOV_II000100	22.9740958	3.478312299	6.604954881	0.151401488
BBOV_III000700	20.52508246	3.406670199	6.024969034	0.165975957
BBOV_II001370	117.6823372	21.98983005	5.351671067	0.186857523
BBOV_I005160	58.84307326	14.5715978	4.038203227	0.247634887
BBOV_IV005640	16.05860815	5.164916596	3.109170856	0.321629157
BBOV_I005885	58.78805667	19.15570372	3.068958339	0.325843459
BBOV_III001295	2.354492652	0.778101658	3.025944783	0.330475297
BBOV_I005925	323.1792347	120.5558705	2.680742409	0.373030992
BBOV_IV000200	1221.015774	33.55945	36.38366462	0.027484862
BBOV_II004170	25.25477425	0.100497771	251.2968606	0.003979357
BBOV_III006070	971.8226855	4.00958644	242.3747935	0.004125842
BBOV_III003100	4179.375093	21.0944595	198.1266737	0.005047276
BBOV_IV001500	736.2579763	6.232070813	118.1401814	0.008464521
BBOV_I005955	156.0360625	2.111219752	73.90801565	0.013530332
BBOV_I005140	122.6569861	2.272128462	53.98329723	0.018524248
BBOV_IV007990	399.8551031	8.592072955	46.5376755	0.021487966
BBOV_II000370	203.6019731	4.45954771	45.65529653	0.021903264
BBOV_III002330	82.99101801	2.13898475	38.79925651	0.02577369
BBOV_III002310	47.30825938	1.381833563	34.23585926	0.02920914
BBOV_I005935	204.0221091	6.004055555	33.98071641	0.029428455
BBOV_III007690	56.20382698	1.679979071	33.45507569	0.029890831
BBOV_II004190	31.41995041	1.005279965	31.25492549	0.031994957
BBOV_I005895	36.94778066	1.235341766	29.90895449	0.033434803
BBOV_I005915	69.19411686	2.391600542	28.93213798	0.03456364
BBOV_III000040	22.04538552	0.841860402	26.18650963	0.038187602
BBOV_I005845	85.75392604	3.346863777	25.622174	0.039028694
BBOV_III000710	24.83032387	0.978632889	25.37246004	0.039412812
BBOV_II004120	62.60907076	2.697046176	23.21394098	0.043077563
BBOV_III007730	100.4983749	4.340448271	23.15391604	0.043189238
BBOV_III007720	52.02941561	2.278964278	22.83029011	0.043801458
BBOV_IV006410	37.49204361	1.667538318	22.48346752	0.044477125
BBOV_I005875	56.76625474	3.27019997	17.3586494	0.057608169
BBOV_II001400	133.9557957	7.782021543	17.21349587	0.058093952
BBOV_I005815	72.0948074	4.198351819	17.17216911	0.058233761
BBOV_IV006400	39.62063076	2.351978021	16.84566369	0.059362458
BBOV_IV000060	20.97555289	1.258803089	16.66309295	0.060012868
BBOV_IV007910	38.04169291	2.348792269	16.19627815	0.06174258
BBOV_I003870	19.33495425	1.212344753	15.94839604	0.06270223
BBOV_I001330	9.327555329	0.692555493	13.46831471	0.074248339
BBOV_II002270	9.706371156	0.770428891	12.59865936	0.079373525
BBOV_III000100	25.84479748	2.285406166	11.30862333	0.088428093
BBOV_I000020	110.8671163	9.939986566	11.1536485	0.089656761
BBOV_II001410	55.15815454	6.181125463	8.923642607	0.112061861
BBOV_IV005680	278.7178629	33.63615638	8.286257791	0.120681739
BBOV_I004520	17.03969061	2.704022755	6.301607696	0.158689663
BBOV_II006780	54.01289193	8.834899055	6.113583369	0.163570191
BBOV_IV002850	2371.499271	427.4573271	5.547920507	0.180247716
BBOV_I001140	10.81090535	2.000911002	5.402991609	0.185082649
BBOV_II002300	2.672564091	0.513720014	5.202374875	0.192219904
BBOV_IV007920	25.37602988	5.163242694	4.914746679	0.203469286
BBOV_II000940	125.7918935	25.9247027	4.852201968	0.206091998
BBOV_I005180	101.9253517	21.35619549	4.772636203	0.209527808
BBOV_I003840	9.184526328	2.050646761	4.478843701	0.223271913
BBOV_II004140	15.36332175	3.952571843	3.886917773	0.257273258
BBOV_I001430	17.06123265	4.433667203	3.848108545	0.259867929
BBOV_II006830	23.9118131	6.226676466	3.8402209	0.260401687
BBOV_I003830	5.60933158	1.611452341	3.480916832	0.287280636
BBOV_II000920	35.70692278	11.62363102	3.071925004	0.32552878
BBOV_I003900	16.74032752	6.142248224	2.72543976	0.366913265
BBOV_I001440	6.681265191	2.610360801	2.559517898	0.390698577
BBOV_II000110	24.00063777	9.819882162	2.444086128	0.409150884
BBOV_II000390	11.18485422	4.944482435	2.262087966	0.442069457
BBOV_IV002860	18.92967276	37.88463844	0.499666185	2.001336153
BBOV_III003110	89.20183852	188.362042	0.473565892	2.111638562
BBOV_III001220	1.72155826	4.18135367	0.411722709	2.42881915
BBOV_I003910	10.52155624	26.5396865	0.396446139	2.522410743
BBOV_IV001510	2.698503508	7.92944196	0.340314428	2.938459015
BBOV_II000910	25.91710922	93.57840916	0.276956078	3.610680819
BBOV_I005805	8.085385626	43.16715254	0.187304122	5.338910788
BBOV_II006790	28.98624709	163.5550893	0.177226201	5.642506559
BBOV_I000030	45.94475751	345.461503	0.132995304	7.519062496
BBOV_III001280	55.72201753	624.213682	0.089267536	11.20228071
BBOV_I005110	5.925071676	74.15213411	0.079904264	12.51497672
BBOV_IV006380	8.428348062	113.9017072	0.073996679	13.51412001
BBOV_III001160	42.67923143	686.2582539	0.06219121	16.07944264
BBOV_I004510	17.47781955	597.9515367	0.029229492	34.21202141
BBOV_I005190	9.67496017	562.8547934	0.017189087	58.17644554
BBOV_III000670	4.364457017	969.2748263	0.004502807	222.0837146

**Table 5:** Differential gene expression of glideosome elements between *B. bovis* blood stages and kinetes

GeneID	norm blood stages	norm kinetes	Fold increase in blood stages	Fold increase in kinetes
BBOV_II006100	5.338814	70978.19	7.52177E-05	13294.74914
BBOV_II006070	1.414999	4259.571	0.000332193	3010.298479
BBOV_II002650	96.21683	164058.8	0.000586478	1705.095019
BBOV_II006080	8.861071	13495.36	0.000656601	1522.994638
BBOV_IV003210	352.6652	26835.99	0.013141504	76.09479332
BBOV_IV008510	325.7201	6390.77	0.050967269	19.62043538
BBOV_IV009790	93.05779	1545.97	0.060193791	16.61300904
BBOV_I000300	1768.456	22238.81	0.079521131	12.57527387
BBOV_II004420	118.6838	1229.741	0.096511253	10.36148606
BBOV_I003490	637.5578	6256.353	0.101905671	9.812996615
BBOV_II006000	509.7894	3351.442	0.152110493	6.574168436
BBOV_II005470	286.0339	670.0373	0.426892467	2.342510298
BBOV_II005940	32.09359	0.249803	128.4757525	0.00778357
BBOV_IV011430	584.8345	5.444342	107.4206191	0.0093092
BBOV_IV011230	537.6968	24.69678	21.77194044	0.045930679
BBOV_II003100	132.8903	6.408557	20.73637991	0.048224425
BBOV_I001630	500.6437	28.21142	17.7461369	0.056350292
BBOV_II005930	150.9749	10.4024	14.51346609	0.068901529
BBOV_II002890	679.6739	61.55152	11.04235702	0.090560376
BBOV_II002630	824.4089	197.8921	4.165952089	0.240041167

## 2 Experimental Design, Materials and Methods

Three splenectomized Holstein calves approximately four months of age and determined to be *Babesia*-free by competitive enzyme-linked immunosorbent assay and PCR [1,2,3] were used for acquisition of *B. bovis* Texas strain by *R. microplus*, La Minita strain, as previously described [1,4]. Approximately 40,000 *R. microplus* larvae were placed under a cloth patch on calves. When ∼1% of the ticks had molted to the adult stage, calves were intravenously inoculated with *B. bovis* stabilate containing 10^7^ infected erythrocytes to synchronize female tick repletion with an ascending parasitemia. Replete female ticks were collected and incubated at 26 °C in 96% relative humidity to allow *B. bovis* development [1,2].

To increase the percent parasitized erythrocytes, blood samples from an acute parasitemia were cultured for 5 days at 3% oxygen and 5% carbon dioxide [5,6]. These *in vitro* cultured *B. bovis* blood stages were centrifuged, media removed, cells suspended in TRIzol (Thermo Fisher Scientific, Waltham, MA) and stored at -80 °C. *Babesia bovis* kinetes were collected from replete female ticks by extraction using pressurized capillary tubing, pooled, concentrated by centrifugation at 3,000 × g for 2 min, suspended in TRIzol and stored at -80 °C. To isolate RNA, the samples were thawed, transferred into Lysing Matrix H tubes and homogenized three times for 30 s at a speed setting of 6.0 m/s (MP Biomedicals, Solon, OH) with cooling of samples on ice for 5 min between runs. Homogenized samples were transferred into microfuge tubes and centrifuged for 1 min at 16,000 x g at 4 °C to remove large particulates. Sample supernatants were transferred to microfuge tubes to which 200 µl of chloroform per ml of TRIzol supernatant was added. The samples were vortexed for 15 s and incubated at room temperature for 3 min. For phase separation, samples were centrifuged at 16,000 x g for 15 min at 4 °C and the upper aqueous phase transferred to a new microfuge tube. Subsequent RNA isolation was accomplished using the RNA Cleanup and Concentrator kit (Zymo Research, Irvine, CA) according to the manufacturer's instructions. In brief, two volumes of RNA binding buffer were added to the aqueous phase, mixed, an equal volume of 100% ethanol added, and mixed again. A volume of 800 µl of each sample was transferred to RNA-25 concentrator column assemblies and the RNA bound to the column matrix by passing the sample through the column by centrifuging at 13,000 x g for 30 s. To the sample-bound column, 400 µl of RNA prep buffer was added and spun at 13,000 x g for 30 s. Samples were washed by adding 800 µl of RNA wash buffer to each column, centrifuged at 13,000 x g for 30 s, washed again with 400 µl of RNA wash buffer, and centrifuged for 2 min at 13,000 x g after the final wash. Columns were transferred to new 1.5 ml tubes and samples eluted by adding 60 µl of nuclease-free water. Samples were treated with TURBO DNase (ThermoFisher Scientific) according to the manufacturer's instructions. Reactions were terminated and samples concentrated using RNA Cleanup and Concentrator RNA-5 or RNA-25 columns as described above. Sample RNA concentrations were determined using a NanoDrop 1000 (ThermoFisher Scientific) and tested for residual DNA by Real-Time SYBR Green PCR targeting of BBOV_II006950. If DNA contamination was observed, samples were re-treated with TURBO DNase as described above until samples were confirmed to be free of DNA. RNA samples were stored at -80 °C. Total RNA was monitored for quality control using the Agilent Bioanalyzer Nano RNA chip (Agilent Technologies, Santa Clara, CA) and NanoDrop absorbance ratios for 260/280nm and 260/230nm. Library construction was performed according to the Illumina TruSeq mRNA (Illumina, San Diego, CA) stranded protocol. Using an input quantity for total RNA within the recommended range, mRNA was enriched using oligo dT magnetic beads. The enriched mRNA was chemically fragmented. First strand synthesis used random primers and reverse transcriptase to make cDNA. After second strand synthesis the ds cDNA was cleaned using AMPure XP beads (Beckman Coulter, Brea, CA) and the cDNA was end repaired and the 3’ ends adenylated. Illumina barcoded adapters were ligated to the ends and the adapter ligated fragments were enriched by nine cycles of PCR. The resulting libraries were validated by qPCR and sized using an Agilent Bioanalyzer DNA high sensitivity chip. The library concentrations were normalized and then multiplexed. The multiplexed libraries were sequenced using paired end 100 cycle chemistry for the HiSeq 2500 (Illumina). The version of HiSeq control software was HCS 2.2.58 with real time analysis software, RTA 1.18.64. Low quality reads were filtered before alignment to the new reference genome using STAR v2.5.2a (2-pass mapping). Counts were generated from alignments for each gene using the Subread feature of Counts v1.6.0. Genes without at least 1 read per million mapped reads across all three samples within a group were removed, data were normalized using the TMM method, and analysed for differential expression significance testing using the generalized linear model likelihood ratio test (GLM LRT) method in edgeR v3.20.9. The false discovery rate (FDR) method was employed to correct for multiple testing and genes were termed differentially expressed if their log Fold Change (logFC) value was greater than or equal to 1 with the FDR set to 5%. Triplicate RNA samples from *B. bovis* blood or kinete stages were used for qPCR. Approximately 100 ng of total RNA from each preparation of parasites was reverse transcribed using a Superscript III™ cDNA Synthesis Kit (ThermoFisher Scientific) following the manufacturer's protocol. Quantitative PCR was performed in triplicate using a Bio-RAD CFX96™ Real-Time PCR Detection System. Reaction volumes were 20 μl using 10 μl SsoFast™ EvaGreen® Supermix (Bio-Rad, Hercules, CA, USA), 1 μl of 500 nM of each primer set, 6 μl of nuclease-free water and 2 μl of a 1:10 dilution of cDNA as template. The conditions consisted of an enzyme activation step of 98 °C for 2 min followed by 40 cycles of 98 °C for 5 s and 55 °C for 5 s. The transcript level of six genes of interest was normalized by dividing the transcript level of the gene of interest with that of the housekeeping genes. To identify suitable “housekeeping” genes, Excel (Microsoft Office 2013) with NormFinder and the comparative delta-Ct method from the RNA-Seq dataset were used to select five genes that had no significant fold changes between the blood and kinete stages [7]. The selected housekeeping genes were then used to normalize transcript levels in the qPCR data.

## 3 Ethics Statement

Animal experiments were conducted with the approval of the Institutional Animal Care and Use Committee of the University of Idaho, Moscow, Idaho, in accordance with institutional guidelines based on the U.S. National Institutes of Health Guide for the Care and Use of Laboratory Animals. Splenectomies were performed under sedation with xylazine and isoflurane inhalation, and all efforts were made to minimize suffering. All animals exposed to an exotic pathogen and ticks were euthanized. These animals were sedated with xylazine, brought to a recumbent position and euthanized by intravenous injection of sodium pentobarbitone. IACUC #2018-16.

## CRediT Author Statement

Massaro Ueti, Wendell Johnson, and Kelly Brayton: Conceptualization. Massaro Ueti, Wendell Johnson, Lowell Kappmeyer, David Herndon, Kathryn Reif: Methodology. Massaro Ueti, Wendell Johnson, Lowell Kappmeyer, David Herndon, Michelle Mousel, Carlos Suarez and Kelly Brayton: Data curation, Writing-Original draft preparation. Massaro Ueti, Wendell Johnson, and Kelly Brayton: Visualization, Investigation. Massaro Ueti: Supervision. Massaro Ueti, Wendell Johnson and Kelly Brayton: Validation of data. Massaro Ueti, Wendell Johnson, Naomi Taus, Olukemi Ifeonu, Joana Silva, Carlos Suarez, and Kelly Brayton: Writing- Reviewing and Editing.

## Declaration of Competing Interest

The authors declare that they have no known competing financial interests or personal relationships which have, or could be perceived to have, influenced the work reported in this article.

## Appendix Supplementary materials

Image, application 1

## References

[1] Howell J.M., Ueti M.W., Palmer G.H., Scoles G.A., Knowles D.P. (2007). Transovarial transmission efficiency of *Babesia bovis* tick stages acquired by *Rhipicephalus* (*Boophilus*) *microplus* during acute infection. J. Clin. Microbiol..

[2] Johnson W.C., Taus N.S., Reif K.E., Bohaliga G.A.R., Kappmeyer L.S., Ueti M.W. (2017). Analysis of stage-specific protein expression during *Babesia bovis* development within female *Rhipicephalus microplus*. J. Proteome Res..

[3] Chung C.J., Suarez C.E., Bandaranayaka-Mudiyanselage C.L., Bandaranayaka-Mudiyanselage C.B., Rzepka J., Heiniger T.J., Chung G., Lee S.S., Adams E., Yun G., Waldron S.J (2017). A novel modified-indirect ELISA based on spherical body protein 4 for detecting antibody during acute and long-term infections with diverse *Babesia bovis* strains. Parasit.Vectors.

[4] Howell J.M., Ueti M.W., Palmer G.H., Scoles G.A. (2007). D.P. Knowles. Persistently infected calves as reservoirs for acquisition and transovarial transmission of *Babesia bovis* by *Rhipicephalus* (*Boophilus*) *microplus*. J. Clin. Microbiol..

[5] Levy M.G., Ristic M. (1980). *Babesia bovis*: continuous cultivation in a microaerophilous stationary phase culture. Science.

[6] J.A. Alvarez, C. Rojas, J.V. Figueroa. Diagnostic tools for the identification of *Babesia* sp. in persistently infected cattle. Pathogens, 8(2019):143. doi:10.3390/pathogens8030143. 10.3390/pathogens8030143 PMC6789608 31505741

[7] Andersen C.L., Jensen J.L., Orntoft T.F. (2004). Normalization of real-time quantitative reverse transcription-PCR data: a model-based variance estimation approach to identify genes suited for normalization, applied to bladder and colon cancer datasets. Cancer Res..

